# Toll-Like Receptor 4 Deficiency Impairs Motor Coordination

**DOI:** 10.3389/fnins.2016.00033

**Published:** 2016-02-16

**Authors:** Jian-Wei Zhu, Yi-Fei Li, Zhao-Tao Wang, Wei-Qiang Jia, Ru-Xiang Xu

**Affiliations:** Affiliated Bayi Brain Hospital, Military General Hospital of Beijing PLA, Southern Medical UniversityBeijing, China

**Keywords:** TLR4, cerebellum, motor coordination, cerebellum-related behaviors, Purkinje cells, Bergmann glia, granule cell, microglia

## Abstract

The cerebellum plays an essential role in balance and motor coordination. Purkinje cells (PCs) are the sole output neurons of the cerebellar cortex and are critical for the execution of its functions, including motor coordination. Toll-like receptor (TLR) 4 is involved in the innate immune response and is abundantly expressed in the central nervous system; however, little is known about its role in cerebellum-related motor functions. To address this question, we evaluated motor behavior in TLR4 deficient mice. We found that TLR4^−∕−^ mice showed impaired motor coordination. Morphological analyses revealed that TLR4 deficiency was associated with a reduction in the thickness of the molecular layer of the cerebellum. TLR4 was highly expressed in PCs but not in Bergmann glia or cerebellar granule cells; however, loss of TLR4 decreased the number of PCs. These findings suggest a novel role for TLR4 in cerebellum-related motor coordination through maintenance of the PC population.

## Introduction

The mammalian cerebellum plays a critical role in motor control and coordination (Schmahmann, 1997; Mauk et al., 2000; Glickstein et al., 2009). PCs, are the sole output neurons of the cerebellar cortex and are indispensable for the execution of its functions (Saywell et al., 2014). Defects in PC function can lead to cerebellar ataxia (Brown and Loew, 2012; Guan et al., 2014; Lucas et al., 2014) and behavioral abnormalities related to balance.

Toll-like receptors (TLRs) are a family of transmembrane pattern recognition receptors that play a crucial role in signal transduction in the innate immune response (Aderem and Ulevitch, 2000). TLRs have been implicated in inflammatory and autoimmune central nervous system diseases (Kerfoot et al., 2004; Chakravarty and Herkenham, 2005) and the brain's response to pathogens and cellular debris (Bsibsi et al., 2002; Bottcher et al., 2003; Maslinska et al., 2004). TLR4 is predominantly expressed by microglia (Lehnardt et al., 2002, 2003) and to a lesser extent by neurons (Zhao et al., 2014) and adult neural progenitor cells (NPCs) (Rolls et al., 2007). It is associated with both neuroprotective (Tahara et al., 2006; Marsh et al., 2009) and neurotoxic (Tang et al., 2008; Abate et al., 2010) effects, neuropathic pain (Lewis et al., 2012), neurodegenerative diseases (Heneka et al., 2014) developmental and adult neuroplasticity (Okun et al., 2011), and adult hippocampal neurogenesis (Rolls et al., 2007); loss of TLR4 increases adult NPC proliferation and neuronal differentiation. However, little is known about the role of TLR4 in motor coordination. We addressed this question in the present study by evaluating motor-related behaviors in TLR4-deficient mice. We found that TLR4 is required for normal motor coordination and the maintenance of the cerebellar PC population. Our findings reveal a previously undescribed role for TLR4 in cerebellum-related motor function.

## Materials and methods

### Mice

Homozygous C57BL/10ScNJ mice (B6.B10ScN-Tlr4^lps−del^/JthJ) (Okun et al., 2012; Lee et al., 2015) were obtained from Jackson Laboratories and were bred with wild type (WT) C57BL/6J mice to obtain heterozygote (TLR4^+∕−^) mice. Homozygous mutants (TLR4^−∕−^) and WT littermates were generated by intercrossing TLR4^+∕−^ mice. Animals were housed under standard laboratory conditions on a 12:12-h light-dark cycle with free access to food and water. The study protocol was approved by the Institutional Animal Care and Use Committee of Affiliated Bayi Brain Hospital, Military General Hospital of Beijing PLA, Southern Medical University.

### Genotyping

Mice were genotyped by PCR using mouse tail genomic DNA and the following forward and reverse primers: TLR4^−∕−^ (5′-GCA AGT TTC TAT ATG CAT TCT C-3′) and (5′-CCT CCA TTT CCA AAT AGG TAG-3′); and WT (5′-ATA TGC ATG ATC AAC ACC ACA G-3′) and (5′-TTT CCA TTG CTG CCC TAT AG-3′). The TLR4^−∕−^ (140 bp products) and WT (390 bp products) allele were determined by PCR under the cycle conditions: 3 min at 94°C, 34 × (30 s at 94°C, 60 s at 55°C, 60 s at 72°C), and 2 min at 72°C using standard PCR reagents according to Jackson Laboratory. PCR products were visualized by electrophoresis on a 1% agarose gel (Figure S1).

### Behavioral tests

**Figure 1:**
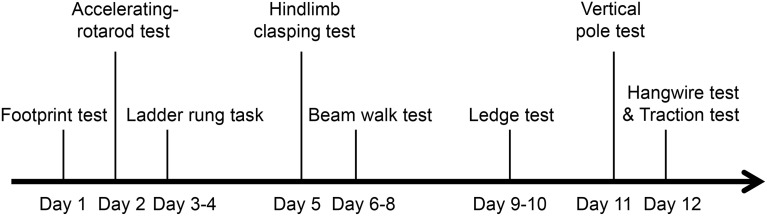
**Experimental paradigm of behavioral tests for assessing motor function**.

Behavioral testing of TLR4^−∕−^ mice (6 months old; six male and six female) and their WT littermates (four male, five female) was carried out during the light phase (between 09:00 and 17:00 h) by investigators blinded to the genotype of the mice. The experimental paradigm for behavioral tests is shown in Figure 1. Each mouse was subjected to each of the following behavioral tests.

#### Footprint test

The fore- and hindpaw were painted with nontoxic red and black ink, respectively. Mice walked through a tunnel (70 cm long, 10 cm wide, 10 cm high) with white paper lining the floor (Clark et al., 1997) to their home cage, which was accessible through a hole. Each mouse underwent three pre-trials followed by three trials. For each of six successive paws prints, a point was marked at the base of the middle toe, and the straight line distance of consecutive ipsilateral hindpaw prints was measured as the stride length; the perpendicular distance from the point on the hindpaw print to the next contralateral hindpaw print was measured as the stride width; and the straight line distance from the hindpaw to the ipsilateral adjacent forepaw was measured as inter-limb coordination. For each measurement, the first and last 10 cm of the prints were excluded. If the mouse stopped in the middle of the tunnel, the trial was repeated.

#### Accelerating-rotarod test

Motor balance and coordination were evaluated with the accelerating-rotarod test as previously described (Wei et al., 2011), with some modifications. Mice were placed on the rotarod facing opposite to the direction of rotation. The initial speed of the rotarod was 4 rpm. After 10 s, the rod was accelerated from 4 to 40 rpm in 4 min and maintained at a constant speed for 1 min. Mice that left the rotarod before acceleration began were placed again on the rod. The time that the mouse remained on the rotarod before falling and rpm at the time of falling were recorded over a maximum observation period of 5 min. Data from three trials were averaged for each mouse.

#### Ladder rung task

The apparatus (Bioseb) was composed of two side walls (90 cm long, 20 cm high) that had 77 plastic bars (7 cm long, 0.5 cm diameter) along the bottom edge and were separated by 5 cm to allow passage of the mouse but prevented it from turning around. The whole apparatus was elevated 20 cm above the ground with a home cage at the end. Mice were tested with regular and irregular ladder patterns. For the former, rungs were spaced at 1-cm intervals; while for the latter, the distance between rungs varied from 1 to 2 cm (Metz and Whishaw, 2002). Each mouse underwent three pre-trials followed by three trials. The number of limb errors (Farr et al., 2006) and crossing time were recorded. Data from three trials were averaged for each mouse.

#### Hindlimb clasping test

Mice were suspended by their tail and the extent of hindlimb clasping was observed for 30 s. If both hindlimbs were splayed outward away from the abdomen with splayed toes, a score of 0 was given. If one hindlimb was retracted or both hindlimbs were partially retracted toward the abdomen without touching it and the toes were splayed, a score of 1 was assigned. If both hindlimbs were partially retracted toward the abdomen and were touching the abdomen without touching each other, a score of 2 was given. If both hindlimbs were fully clasped and touching the abdomen, a score of 3 was assigned (Guyenet et al., 2010; Li et al., 2012).

#### Beam walk test

A narrow beam (70 cm long, diameter 1 cm) was placed horizontally 60 cm above the platform surface, with one end fixed on the platform and the other linked to a closed, bright goal box (25 cm^2^). Mice were tested after 2 days training. Briefly, mice were habituated to the goal box for 3 min and then placed at a distance of 10 cm from the goal box on the beam. Once the mouse could traverse the beam to reach the goal box without difficulty, they were placed at increasing distances away from the goal box (20, 30, 50, and 70 cm) and trained to traverse the beam for three or four consecutive trials from each starting point (Quinn et al., 2007). The time taken for the mouse to cross the full length of the beam to the goal box was recorded for a maximum time of 120 s. If the mouse fell before reaching the goal box, the time was recorded as 120 s. The time spent in a frozen posture and number of paw slips were also noted. The number of times the paws-clasped the beam during walking was also recorded. Data from three trials were averaged for each mouse.

#### Ledge test

The ledge test for evaluating balance and coordination was carried out as previously described (Guyenet et al., 2010), with a minor modification. Mice were placed on a ledge (90 cm long, 0.5 cm wide, 20 cm high), and paw placement and forward movement as it walked along the ledge were observed. The time spent to cross the ledge and number of paw slips were also recorded. Mice that left the ledge were excluded.

#### Vertical pole test

Motor coordination and balance were assessed with the vertical pole test. A plastic pole (50 cm high, diameter 1 cm) was placed vertically on a soft platform (Montana et al., 2011); mice were placed at the top of the pole and the time taken to turn around and climb down the pole was recorded. A maximum time of 120 s was given to complete the task. If the mouse fell from the top of the pole, a time of 120 s was recorded. Each mouse underwent three trials at 20-min intervals. Data from three trials were averaged for each mouse.

#### Hangwire test

Balance and grip strength were evaluated with this test. Briefly, mice were hung upside down on a wire screen (12 × 12 mm grids) (Maejima et al., 2013) 50 cm above a mouse cage. The time until the mouse fell off the screen and into the cage was recorded. Mice that did not fall off within the 120 s trial period were removed and assigned a maximal time of 120 s.

#### Traction test

Grip strength was measured with the traction test. The mouse was allowed to grasp a bar (1 mm diameter) with their forepaws and was slowly pulled backward by the tail. The maximum tension (in grams) before the mouse released the bar was recorded and normalized to body weight (Van Damme et al., 2003). Each mouse underwent three trials with a 5-min recovery time between trials. Data from three trials were averaged for each mouse.

### Histological analysis and immunohistochemistry

Mice (6 months old, 22.5–26.8 g) were anesthetized with 3.6% chloral hydrate and transcardial perfusion was carried out with phosphate-buffered saline (PBS) followed by 4% (4 g in 100 ml) paraformaldehyde (PFA) in 0.1 M PBS (pH 7.4) (5 ml/g body weight). The brain was dissected and fixed in 4% PFA in 0.1 M PBS (pH 7.4) overnight at 4°C, then cryoprotected in 30% sucrose in PBS for 48 h at 4°C. Sagittal sections (25 μm) were cut with a cryostat and mounted onto glass slides. Cryosections were stained with hematoxylin and eosin (H&E). For immunohistochemistry, frozen sections were air-dried and rinsed with PBS before incubation in 0.3% Triton X-100 in PBS for 30 min. Sections were blocked (10% bovine serum albumin and 0.3% Triton X-100 in PBS) for 1 h at room temperature before primary antibody treatment. Primary antibody and their dilutions were anti-TLR4 (1:200; Abcam, ab13556), anti-Calbindin (1:1000; Swant, 300), anti-Sox2 (1:100; Santa Cruz, sc-17320), anti-NeuN (1:200; Abcam, ab104224), anti-Iba1 (1:400; Abcam, ab5076). The sections were then washed (three times) with PBS containing 0.3% Triton X-100 for 30 min and incubated with primary antibody for 16 h at room temperature, followed by Alexa Fluor secondary antibody (1:1000; Invitrogen, Carlsbad, CA, USA) for 2 h at room temperature. After three washes with PBS, sections were mounted with medium containing DAPI (Vector Laboratories, Burlingame, CA, USA) and visualized by confocal microscopy.

### Quantitative analysis

The cerebellar volume was determined as the sum of volumes in consecutive H&E- stained sagittal sections (100 μm) from each mouse, as previously described (Isaacs and Abbott, 1995; Manninen et al., 2014). Briefly, the cerebellar surface area were estimated with ImageJ software (National Institutes of Health, Bethesda, MD, USA) using the grid point (10 × 10 mm) counting method (Gundersen, 1980). The entire cerebellar volume was estimated using the formula: Volume = Σ*P* ^*^ *t* ^*^α (*p*), where Σ*P* is the total number of grid points counted in all sections, *t* is the thickness of the sections, and α (*p*) is the area associated with each point. The area of the cerebellum was determined by measuring six H&E-stained sagittal sections along the anterior- posterior extent of the cerebellum for each mouse. An outline of the cerebellum was measured using ImageJ software. The thickness of lobule VIII of the molecular layer (ML) was similarly measured. Briefly, we chose three sides of the ML in lobule VIII and drew a line through the center and perpendicular to both the PC layer (PL) and ML surface on each side. The line length between the PL and ML surface was regarded as the thickness of the ML. Data from three sides were averaged for each section. Calbindin-positive PCs in a 200-μm line along lobule VIII of the entire PL were counted in six sagittal sections from identical levels in each mouse. Ionized calcium-binding adapter molecule (Iba1)-positive microglia in a 300 × 300 μm^2^ area of lobules I–IV in each section were counted and the percent stained area was also measured with ImageJ software in each one z-slice image. NeuN-expressing granule cells in the granule cell layer (GCL) in a 100 × 100 μm^2^ area of lobule III were also counted in each section.

### Statistical analysis

Data are represented as mean ± SEM and were analyzed by the Student's two-tailed unpaired *t*-test using SPSS v.18.0 software (SPSS Inc., Chicago, IL, USA). *P* < 0.05 was considered as statistically significant.

## Results

### TLR4 deficiency decreases balance and motor control

**Figure 2:**
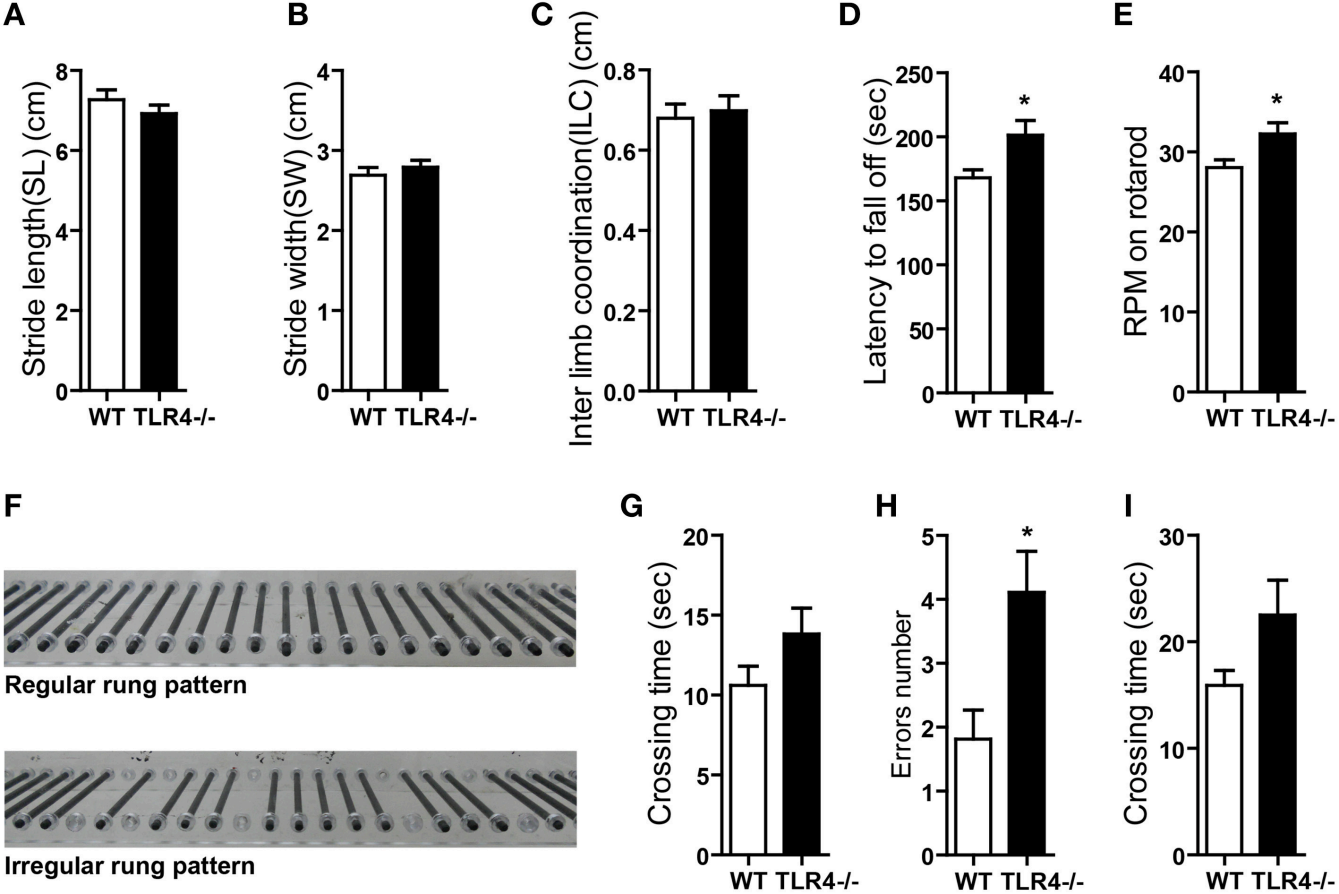
**TLR4 deficiency impairs balance and motor control. (A–C)** TLR4^−∕−^ and WT mice showed similar stride length **(A)**, stride width **(B)**, and inter-limb coordination **(C)** in the footprint test. **(D,E)** TLR4^−∕−^ mice showed better performance than WT mice in the accelerating- rotarod test; the time taken to fall off of the rotarod **(D)** and maximum rpm before falling **(E)** were greater in mutants. **(F)** Photograph illustration of regular and irregular ladder rung patterns in the ladder rung test. **(G)** Crossing time was longer for TLR4^−∕−^ as compared to WT mice with the regular rung pattern. **(H,I)** Number of errors **(H)** and crossing time **(I)** were increased in TLR4^−∕−^ as compared to WT mice with the irregular rung pattern. Data are presented as mean ± SEM (*n* = 9–12 mice/group). ^*^P < 0.05.

The role of TLR4 in motor control was assessed by analyzing gait with the footprint test. There were no differences in stride length (Figure 2A) [*t*_(19)_ = 1.068, *p* = 0.299] or stride width (Figure 2B) [*t*_(19)_ = −0.775, *p* = 0.448] or interlimb coordination (Figure 2C) [*t*_(19)_ = −0.358, *p* = 0.724] between TLR4^−∕−^ and WT mice. To assess abnormalities in motor function, mice were tested on an accelerating- rotarod test. Consistent with previous studies (Okun et al., 2012), TLR4^−∕−^ mice showed better performance than their WT littermates, with longer times on the rotarod (Figure 2D) [*t*_(19)_ = −2.303, *p* = 0.033] and higher rpm before falling off the rod (Figure 2E) [*t*_(19)_ = −2.310, *p* = 0.032]. However, mutants tended to bend down and remain close to rather than stand on the rod and face front, which was observed in WT mice (Video 1). TLR4^−∕−^ mice also exhibited more rapid limb movement while running on the rotarod than WT mice. Given that the weight of the mouse can influence the results of the rotarod test (Brooks and Dunnett, 2009), we compared the weights of TLR4^−∕−^ and WT mice and found them to be similar (Figure S2C) [*t*_(19)_ = 0.953, *p* = 0.353]. Motor balance was evaluated with the ladder rung test (Farr et al., 2006); there was no difference in the number of limb errors with the regular rung pattern (data not shown) between the two groups, but crossing time was longer in the mutants (Figure 2G) [*t*_(19)_ = −1.507, *p* = 0.148]. To test whether the longer time compensated for fewer limb errors, mice underwent the same test but with irregularly spaced bars (Figure 2F). The number of limb errors (Figure 2H) [*t*_(19)_ = −2.723, *p* = 0.013] and crossing time (Figure 2I) [*t*_(19)_ = −1.661, *p* = 0.113] were increased for TLR4-deficient mice. These results indicate that TLR4 deficiency affects balance and motor control.

### TLR4 deficiency impairs motor coordination

The cerebellum plays a critical role in the control of balance and motor coordination. We therefore compared cerebellum-related behaviors between TLR4^−∕−^ and WT mice.

**Figure 3:**
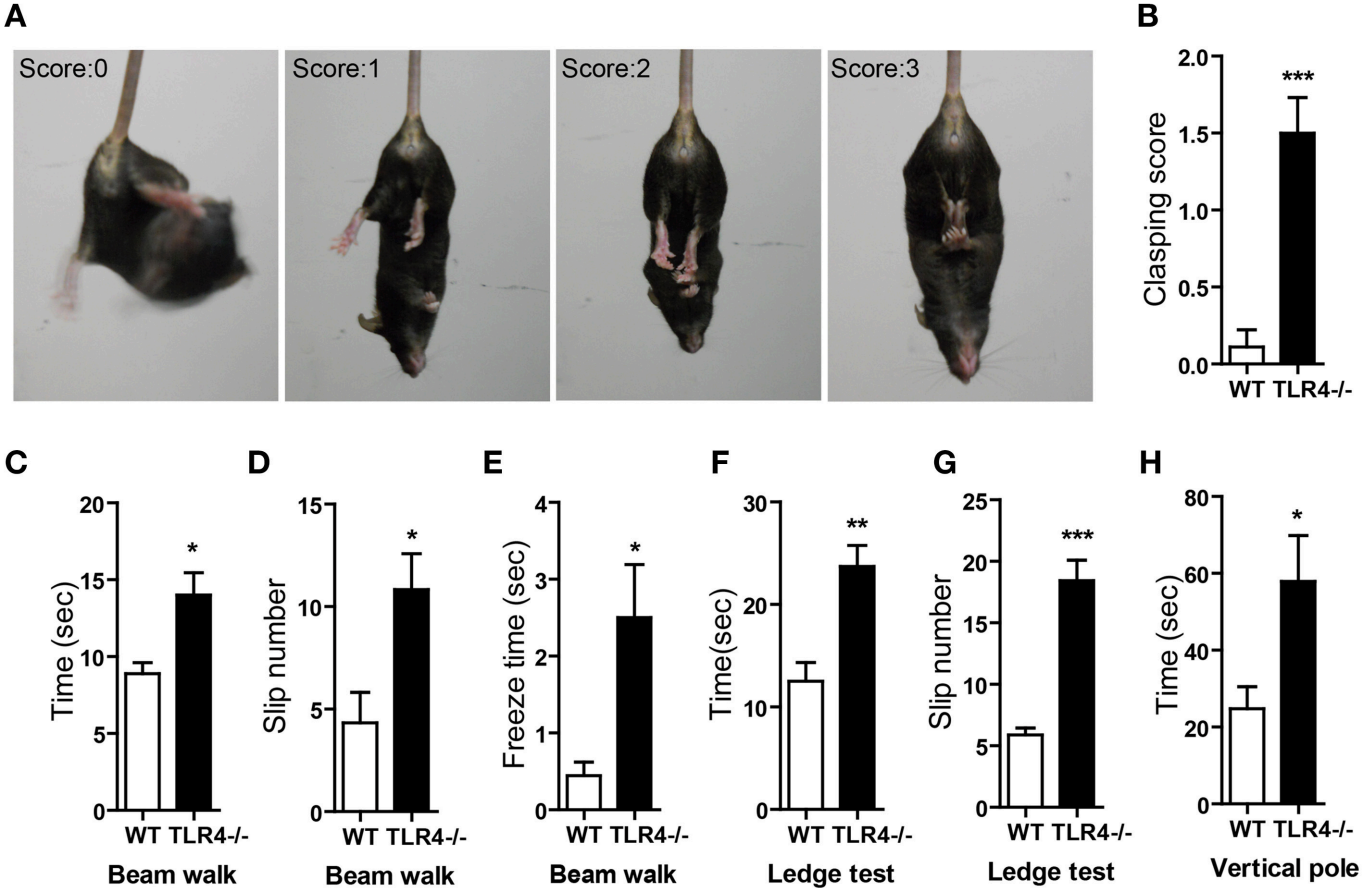
**TLR4 deficiency impairs motor coordination. (A)** Representative posture and scoring in the hindlimb clasping test. **(B)** Quantitative analysis the hindlimb clasping scores; TLR4^−∕−^ mice showed higher scores than WT mice. **(C–E)** Results of the beam walk test. TLR4^−∕−^ mice took longer to cross the beam **(C)**, had a greater number of slips **(D)**, and exhibited longer freeze times **(E)** during beam crossing than WT mice. **(F,G)** Results of the ledge test. TLR4^−∕−^ mice took longer to cross the ledge **(F)** and had a greater number of slips **(G)** than WT mice when walking along the ledge. **(H)** Results of the hangwire test showed that TLR4^−∕−^ mice took longer to turn around and climb down the wire than WT mice. Data are presented as mean ± SEM (*n* = 9–12 mice/group). ^*^P < 0.05, ^**^P < 0.01, ^***^P < 0.001.

The degree of clasping in the hindlimb clasping test is regarded as an indicator of the severity of motor dysfunction (Chou et al., 2008; Takahashi et al., 2009). WT mice showed a normal extension reflex in the hindlimbs and used body torsion to try to grab their tails when suspended in the air; however, hindlimb clasping was observed in TLR4^−∕−^ mice (Figure 3A and Video 2), which showed higher scores in the clasping test than their WT counterparts (Figure 3B) [*t*_(19)_ = −4.888, *p* < 0.001]. We also used the beam walk test to assess motor coordination. A longer time was taken by mutants than by WT mice to cross the beam (Figure 3C) [*t*_(19)_ = −2.847, *p* = 0.01]; moreover, the number of paw slips (Figure 3D) [*t*_(19)_ = −2.711, *p* = 0.014] and freezing time (Figure 3E) [*t*_(19)_ = −2.516, *p* = 0.013] during crossing were greater in the mutants (Video 3). The results of the ledge test revealed impaired motor coordination in TLR4^−∕−^ mice, as evidenced by their difficulty with paw placement along the ledge and limbs slips during forward movement (Video 4). The mutants also took longer to cross the ledge (Figure 3F) [*t*_(13)_ = −4.076, *p* = 0.001] and had more paw slips (Figure 3G) [*t*_(13)_ = −7.483, *p* < 0.001] during crossing than WT mice. In the vertical pole test, TLR4^−∕−^ mice took longer to turn around and climb down the pole than WT mice (Figure 3H) [*t*_(19)_ = −2.257, *p* = 0.024].

Since motor coordination is influenced by muscle strength, we compared the muscle strength of TLR4^−∕−^ and WT mice with two tests. Forelimb strength was assessed by recording maximum grip strength before the mouse released the bar in the traction test; there was no difference between the two groups (Figure S2A) [*t*_(19)_ = −0.757, *p* = 0.458]. Fore- and hind-limb strength was evaluated with the hangwire test; the maximum time that mice held onto the inverted wire was similar between TLR4^−∕−^ and WT mice (Figure S2B) [*t*_(19)_ = −1.235, *p* = 0.232]. These results indicate that TLR4 deficiency leads to loss of motor coordination. Sexual dimorphism has been previously described in the cerebellum (Nguon et al., 2005; Lentini et al., 2013); we therefore analyzed the effect of sex on behavior and found no differences in motor performance between male and female mice. There was also no interaction between sex and genotype (data not shown).

### ML thickness is decreased by loss TLR4

**Figure 4:**
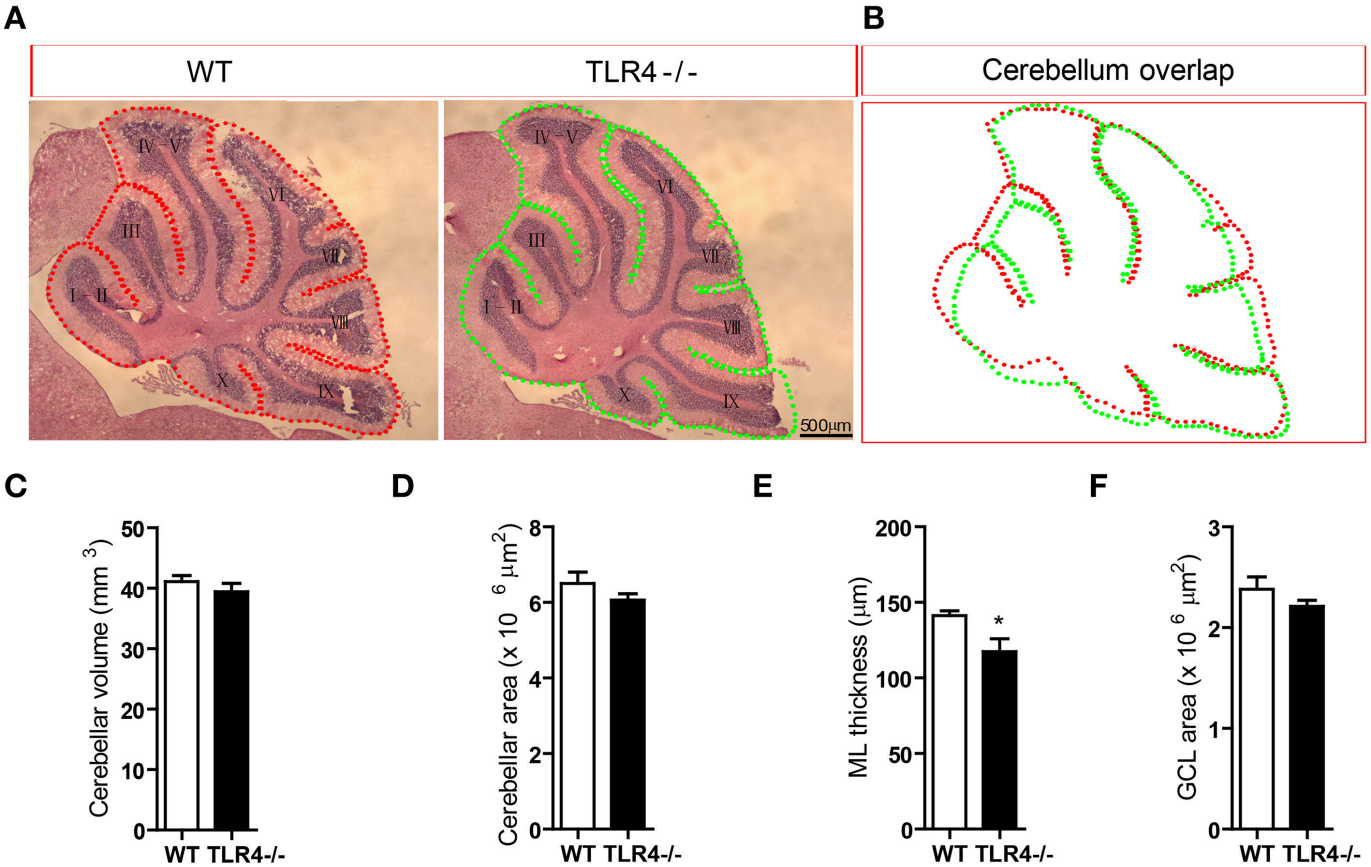
**Loss of TLR4 leads to reduced ML thickness in the adult cerebellum. (A)** There were no differences in lamination and foliation pattern between TLR4^−∕−^ and WT mice, as assessed by H&E staining. **(B)** Cerebellar area did not differ between TLR4^−∕−^ and WT mice. **(C–F)** Quantitative analysis of cerebellar volume **(C)**, area **(D)**, ML thickness **(E)**, and GCL area **(F)** in adult TLR4^−∕−^ and WT mice. Scale bar = 500 μm in **(A)**. Data are presented as mean ± SEM (*n* = 4 mice/group). ^*^P < 0.05.

To further clarify the role of TLR4 in motor coordination, we examined H&E-stained sagittal sections to assess the gross morphology of the cerebellum. There were no differences in lamination or foliation patterns between TLR4^−∕−^ and WT mice (Figure 4A); moreover, the overall size of the cerebellum did not differ between mutant and WT mice (Figure 4B), as evidenced by comparisons of volume (Figure 4C) [*t*_(6)_ = 0.87, *p* = 0.418] and area (Figure 4D) [*t*_(6)_ = 1.292, *p* = 0.244]. There was no effect of sex on cerebellar volume and area (data not shown). However, the thickness of the cerebellar ML was reduced in TLR4^−∕−^ as compared to WT mice (Figure 4E) [*t*_(6)_ = 2.593, *p* = 0.041], whereas the GCL had similar areas in the two groups (Figure 4F) [*t*_(6)_ = 1.244, *p* = 0.260]. These results indicate that the loss of TLR4 results in a decrease in ML thickness.

### TLR4 is highly expressed in cerebellar PCs

**Figure 5:**
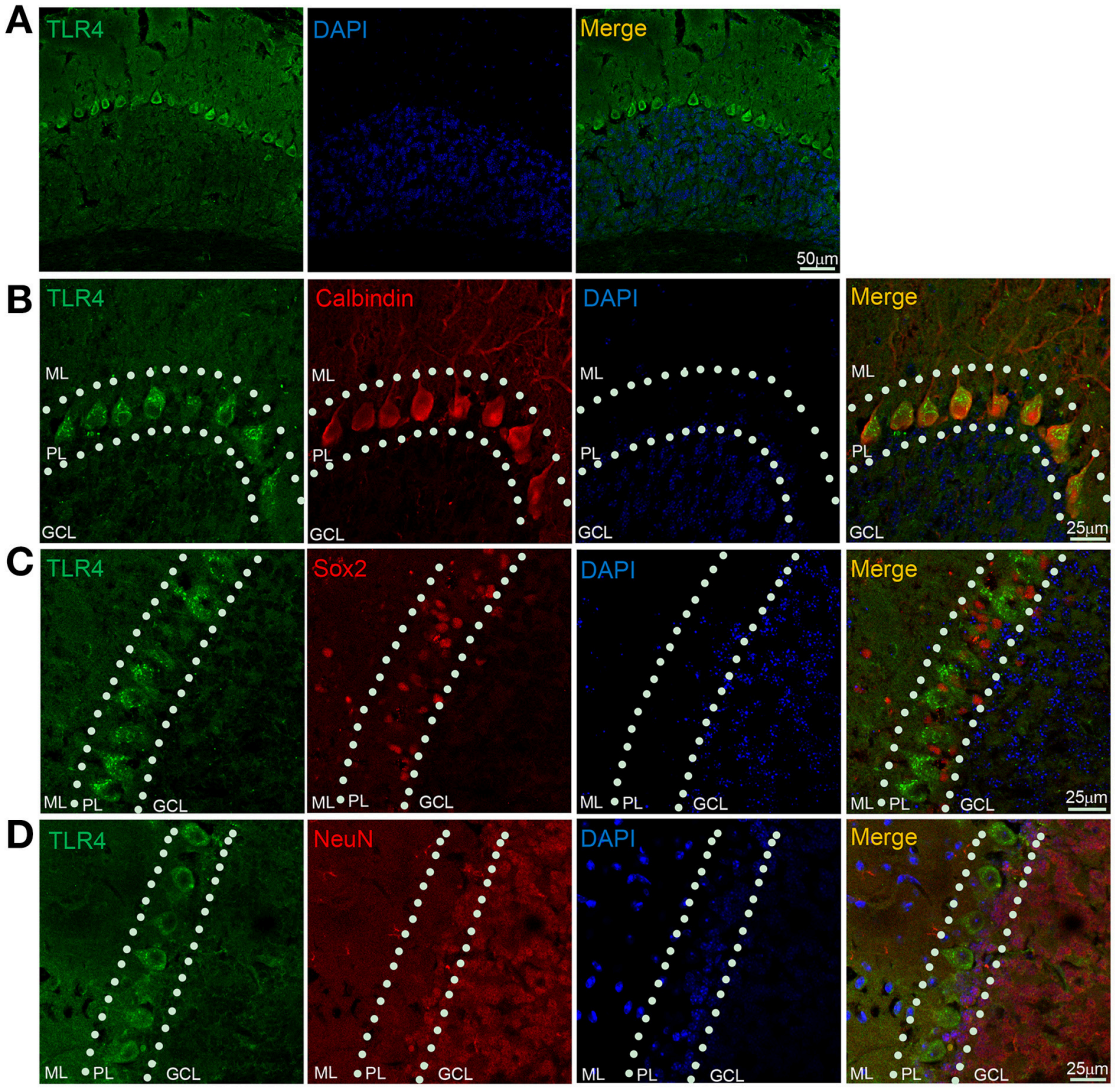
**TLR4 is highly expressed in cerebellar PCs. (A)** TLR4 expression was detected in the PL. **(B)** Immunohistochemical analysis of calbindin revealed colocalization with TLR4 in PCs. **(C)** TLR4 expression was not detected in Sox2-positive BG in the PL. **(D)** NeuN-positive GCs located in the GCL and PL (dashed lines) did not express TLR4. Scar bar = 50 μm in **(A)**, 25 μm in **(B,C,D)**. ML, molecular layer; PL, Purkinje cell layer; GCL, granule cell layer.

We examined TLR4 expression in the cerebellum by immunohistochemistry. TLR4 was highly expressed in the PL (Figure 5A) but no specific immunoreactivity was observed in the TLR4^−∕−^ cerebellum (Figure S3A), although a weak signal was detected in the ML and GCL. A similarly weak signal was also present in the ML and GCL in the section without TLR4 antibody treatment (Figure S3B). Moreover, TLR4 colocalized with the PC marker calbindin (Huang et al., 2014), confirming specific expression in PCs (Figure 5B). Sex-determining region Y-box (Sox2)-positive Bergmann glia (BG) are a specialized type of astrocyte located in the cerebellar PL (Alcock et al., 2007; Shiwaku et al., 2013), with their radial processes spanning the entire ML (Ichikawa-Tomikawa et al., 2012). We found that TLR4 did not colocalize with Sox2 (Figure 5C) or neuronal nuclei (NeuN) (Figure 5D), a GC marker. Since previous study reported that TLR4 is expressed by microglia (Lehnardt et al., 2002, 2003; Zhao et al., 2014), we verified the expression pattern of Iba1, a microglia marker (Ito et al., 1998; Bachstetter et al., 2015; Slusarczyk et al., 2015) and found that it was mostly expressed in the white matter, GCL, and ML (Figure S4C). There were few microglia expressing TLR4 (Figures S3C,D). These results indicate that TLR4 is highly expressed in PCs but not in BG, GCs in the cerebellum, suggesting that the loss of motor coordination in TLR4-deficient mice is associated with PC dysfunction.

### Loss of TLR4 decreases PC number

**Figure 6:**
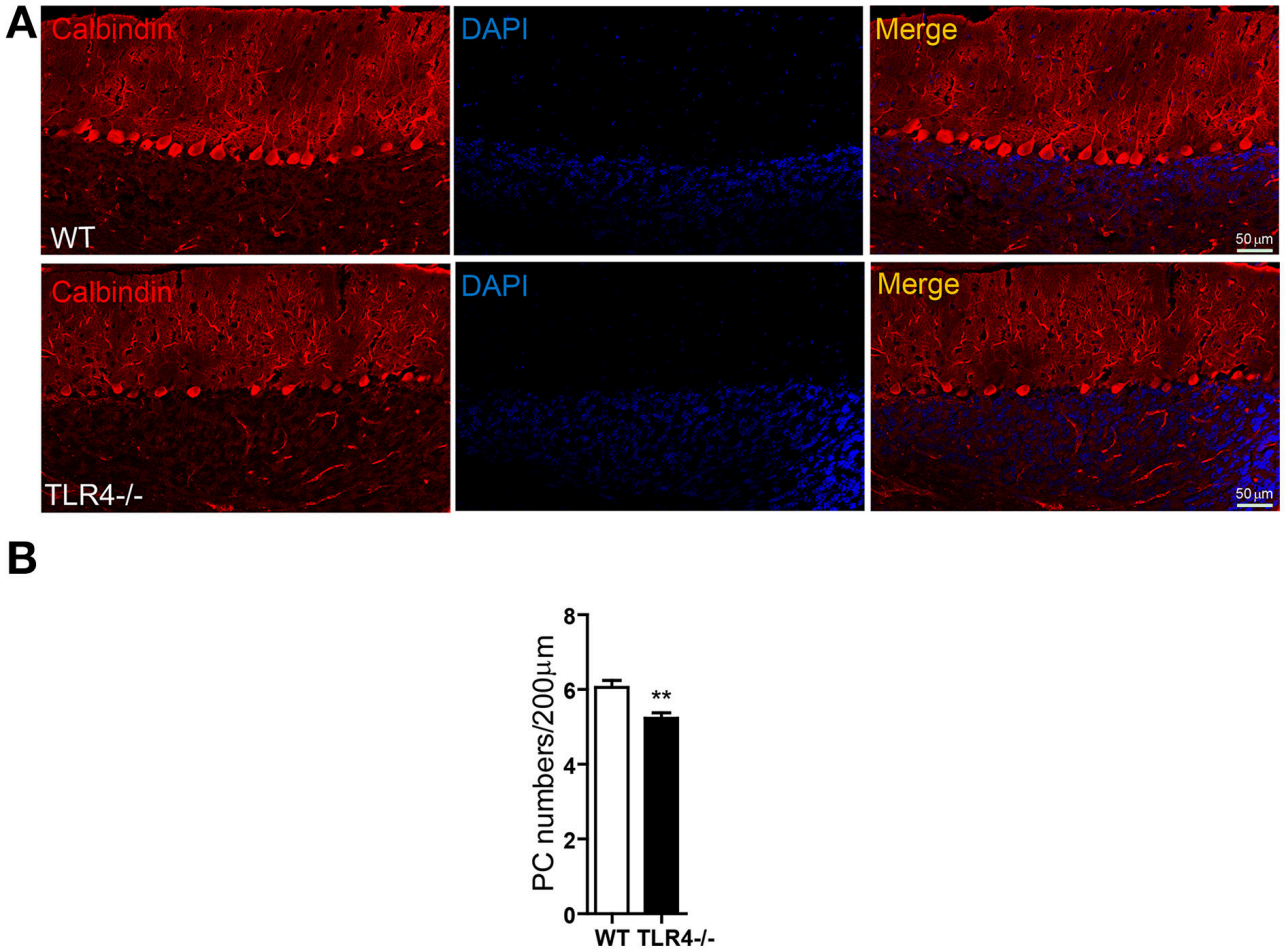
**TLR4 deficiency decreases the number of PCs in adult cerebellum. (A)** PCs were identified by immunolabeling with an anti-calbindin antibody. PC somata were aligned in the PL, with their dendritic arbor spanning the whole ML. **(B)** Quantitative analysis of the number of calbindin-positive PCs in the PL. PC density was lower in TLR4^−∕−^ than in WT mice. Scale bar = 50 μm. Data was presented as mean ± SEM (*n* = 5 mice/group). ^**^P < 0.01.

Given the high expression levels of TLR4 in PCs, we visualized the morphology of PCs by immunohistochemical analysis of calbindin expression and found that their somata were located in the PL and their dendritic arbor spanned the entire ML in both TLR4^−∕−^ and WT mice (Figure 6A), with no obvious differences between the two groups. However, the density of cerebellar PCs was reduced in mutants as compared to WT mice (Figure 6B) [*t*_(8)_ = 3.502, *p* = 0.008], while no differences were observed in the number of NeuN-positive GCs (Figures S4A,B) [*t*_(3)_ = 0.623, *p* = 0.567] or Iba1-positive microglia (Figures S4C,D) [*t*_(3)_ = 0.989, *p* = 0.379]. In addition, the area of Iba1 staining in the cerebellum was similar in the two groups (Figure S4E) [*t*_(4)_ = −0.455, *p* = 0.673]. These results suggest that the loss of motor coordination associated with TLR4 deficiency is due to a depletion of PCs in the cerebellum.

## Discussion

TLR4 is a critical component not only of the innate immune system but also the central nervous system, and is implicated in the regulation of neuropathic pain (Lewis et al., 2012) as well as in neurodegenerative diseases (Heneka et al., 2014). TLR4 also protects hippocampal neurons from ischemic injury via attenuation of the inflammatory response (Hua et al., 2007), and is involved in neuroplasticity (Okun et al., 2011) and neurogenesis (Rolls et al., 2007). TLR4 deficiency enhances adult NPC proliferation and neuronal differentiation, as well as hippocampus-dependent learning and memory. The results of the present study reveal a novel role for TLR4 in cerebellum-related motor coordination.

TLR4^−∕−^ mice exhibited abnormal balance and motor coordination. The footprint test revealed that stride length and width as well as inter-limb coordination were similar between mutant and WT mice, which was likely due to the higher speed of TLR4^−∕−^ mice (Okun et al., 2012), a compensatory mechanism that also improved their performance in the accelerating-rotarod test as we observed and others have reported (Okun et al., 2012). TLR4^−∕−^ mice were bent forward such that their abdomen rested on the rotarod; this likely lowered the body's center of gravity and increased step frequency, which prevented them from falling. TLR4 has been implicated in major depressive disorder (Gárate et al., 2011; Hines et al., 2013; Kéri et al., 2014), which is linked to stress-induced activation of the hypothalamus-pituitary-adrenal axis (Liu et al., 2014). Given that TLR4 also activates this axis (Liu et al., 2014), it is possible that TLR4^−∕−^ mice in the rotarod test were in a state of stress, which could account for their higher step frequency on the rotarod. TLR4^−∕−^ mice in a non-stressed state were able to regulate their steps by slowing down to avoid slipping in the ladder rung test with the regular rung pattern, when the ladder was stationary and close to the ground. However, impaired motor control and balance were observed when the difficulty was increased with the irregular rung pattern, demonstrating the limited effects of this compensatory mechanism.

TLR4-deficient mice exhibited various cerebellum-related motor defects. In addition to abnormal hindlimb clasping behavior, during forward movement in the beam walk or ledge tests, mutants had difficulty with proper limb placement and usually clasped the beam or ledge from the side rather than placing their paws on the surface. Balance and motor coordination depend on proper limb placement, especially when walking on a narrow surface (Metz and Whishaw, 2002). The results of the vertical pole test also revealed impaired motor coordination in TLR4-deficient as compared to WT mice.

Balance and motor coordination are controlled not only by the central nervous system (i.e., the cerebellum), but also depend on peripheral skeletal muscle strength (Zhang et al., 2014). We determined with the traction and the hangwire tests that TLR4^−∕−^ and WT mice had comparable muscle strength. Furthermore, the two groups showed similar body weight, indicating that the loss of motor coordination resulting from TLR4 deficiency was not the result of neuromuscular dysfunction or weight changes.

PCs are the sole output neurons of the cerebellar cortex and are critical for cerebellar information processing (De Schutter and Steuber, 2009; Popa et al., 2012) and execution of motor functions (Fu et al., 1997; Ebner et al., 2011); their dysfunction can result in loss of motor coordination (Donald et al., 2008; He et al., 2014; Bosch et al., 2015) and cerebellar ataxia (Walter et al., 2006; Wang et al., 2015). We found that TLR4 was highly expressed in PCs but not in BG and GCs consistent with the reported expression patterns of TLR4 in other species (Ansari et al., 2015). We also found that loss of TLR4 was associated with a reduction in PC number and ML thickness. These results suggest that TLR4 is essential for maintaining PC survival and functioning; however, the possibility that downstream effectors or signaling pathways are involved cannot be excluded, since we observed no obvious motor impairment in younger TLR4^−∕−^ mice (3 months) (data not shown). This may be explained by a failure to maintain the PC population with increasing age in the absence of TLR4, leading to PC death and consequent thinning of the ML. In the mammalian cerebellum, the progressive retraction of dendritic arbor from PC death leads to a thinner ML with increasing age (Rogers et al., 1984; Dlugos and Pentney, 1994; Hadj-Sahraoui et al., 2001; Zhang et al., 2006); this is similar to the reduction in PC number accompanied by thinning of the ML in the cerebellum of TLR4^−∕−^ mice. It is therefore likely that TLR4 is involved in this process. However, the precise mechanism remains to be elucidated. The control of motor balance and coordination by the cerebellum depends on integral neural circuitry and precise information processing (Ito, 2006; Dean et al., 2010; D'Angelo, 2011). PCs play an important role in building cerebellar circuitry by integrating input from parallel and climbing fibers (Tanaka, 2009) and processing and sending this information to vestibular and deep cerebellar nuclei for proper execution of cerebellar functions (Chan-Palay et al., 1979; Steuber and Jaeger, 2013). The impaired motor coordination exhibited by TLR4^−∕−^ mice is therefore attributable to a loss of PCs.

PCs are GABAergic neurons that originate from NPCs in the ventricular zone during embryogenesis (Carletti and Rossi, 2008). TLR4 is expressed in NPC during embryonic stages and its expression level is maintained throughout adulthood (Lathia et al., 2008; Kaul et al., 2012), suggesting that it is important for the development and maturation of PCs during neurogenesis as well as their survival. In a previous study (Lehnardt et al., 2002, 2003; Zhao et al., 2014), TLR4 expression was mainly detected in microglia. There were few microglia expressing TLR4 in our study. TLR4 is required by microglia for activation of neuroimmunity (Lewis et al., 2012; Heneka et al., 2014) and neuroprotection under pathological conditions (Tahara et al., 2006; Marsh et al., 2009). It is possible that TLR4 expression would be upregulated in cerebellar microglia upon injury; however, our data indicate that the number of microglia and percent area was similar between TLR4-deficient and WT cerebellum. PC degradation resulting from TLR4 deficiency is likely a chronic and complex process that increases with age; similarly, microglia activation in the cerebellum of TLR4^−∕−^ mice may also occur over a long-term, as distinguished from the acute activation that occurs upon injury (Neumann et al., 2009).

## Author contributions

Project design come from Prof. R-X Xu and J-W Zhu, the primary works come from J-W Zhu and we gratefully acknowledge the testing assistance in behavioral test from Y-F Li, in data collection and analysis from Z-T Wang and in immunohistochemistry from W-Q Jia. Manuscript was written by J-W Zhu and revised by Prof. R-X Xu.

### Conflict of interest statement

The authors declare that the research was conducted in the absence of any commercial or financial relationships that could be construed as a potential conflict of interest.

## Supplementary material

Figure S1 **Genotyping of TLR4^−∕−^ and WT mice**. Offspring from the TLR4^±^ intercross were genotyped. PCR amplification was carried out using tail genomic DNA from WT (lane 1), TLR4^±^ (lane 2), and TLR4^−∕−^ (lane 3) mice; H_2_O (lane 4) was used as a control.

Figure S2 **Loss of TLR4 has no effect on muscle strength. (A)** TLR4^−∕−^ and WT mice showed similar limb strength, as measured by the traction test. **(B)** Limb strength was evaluated with the hangwire test and was comparable between TLR4^−∕−^ and WT mice. **(C)** TLR4 deficiency had no effect on body weight. Data are presented as mean ± SEM (*n* = 9–12 mice/group).

Figure S3 **TLR4 is expressed in the PL of the cerebellum and in a small number of microglia. (A)** Immunohistochemical analysis revealed no TLR4 expression in the PL of TLR4^−∕−^ cerebella. **(B)** Weak fluorescence in other layers was attributed to background immunoreactivity. **(C)** TLR4 expression was also detected in Iba1-positive microglia, as shown by arrows in the high-magnification image **(D)**. Scale bar = 100 μm in **(A,B)**, 25 μm in **(C)**, 10 μm in **(D)**. TLR4 Ab, TLR4 antibody.

Figure S4 **TLR4 deficiency has no effect on GC and microglia numbers. (A)** NeuN-expressing GCs in the GCL, with a high-magnification image **(A**′**)**. **(B)** Quantitative analysis of GC density revealed no difference between the two groups. **(C)** Iba1-positive microglia localized in the ML and GCL in both TLR4^−∕−^ and WT mice. Microglia morphology was similar between the two groups, as shown in the high-magnification image **(C**′**)**. **(D,E)** Quantitative analysis of cerebellar microglia showed no difference in microglia number **(D)** or percent area of microglia **(E)** between the two groups. Scale bar = 100 μm in **(A,C)**, 25 μm in **(A′,C′)**. Data are presented as mean ± SEM (*n* = 3 mice/group).

Video 1 **Performance in the accelerating -rotarod test**. TLR4^−∕−^ mice (right) tended to bend down and stay close to the rod as compared to WT mice, which stood on the rod with head toward the front (left) during rod acceleration. Step frequency was higher in mutants than in WT mice.

Video 2 **Hindlimb clasping posture in the hindlimb clasping test**. TLR4^−∕−^ mice (right) showed typical hindlimb clasping posture, whereby both hind-limbs were fully clasped and touching the abdomen after an initial period in which their hind-limbs were splayed outwards away from the abdomen, which was accompanied by body torsion. WT mice (left) exhibited a normal hindlimb extension reflex but maintained this position, with hind-limbs splayed outwards away from the abdomen, splayed toes, and constant body torsion. These results suggest cerebellar ataxic-like behavior in mutants.

Video 3 **Impaired motor coordination in the beam walk test**. TLR4^−∕−^ mice (right) walked slowly and exhibited frequent limb slips on the beam as compared to WT mice (left). Mutants had difficulty maintaining their hind-limbs on the beam and clasping it while walking forward.

Video 4 **Limb placement and forward movement in the ledge test**. TLR4^−∕−^ mice (right) placed their limbs close to the side walls of the ledge instead of on the ledge surface like WT mice (left) when walking along the ledge. TLR4^−∕−^ mice exhibited a slower walking pace and showed a greater number of limb slips than WT mice.
