# Supplementary figures and images for: Toll-Like Receptor 4 Deficiency Impairs Motor Coordination
Source: Front Neurosci. 2016 Feb 16;10:33. doi: 10.3389/fnins.2016.00033 (PMC4754460; doi:10.3389/fnins.2016.00033)

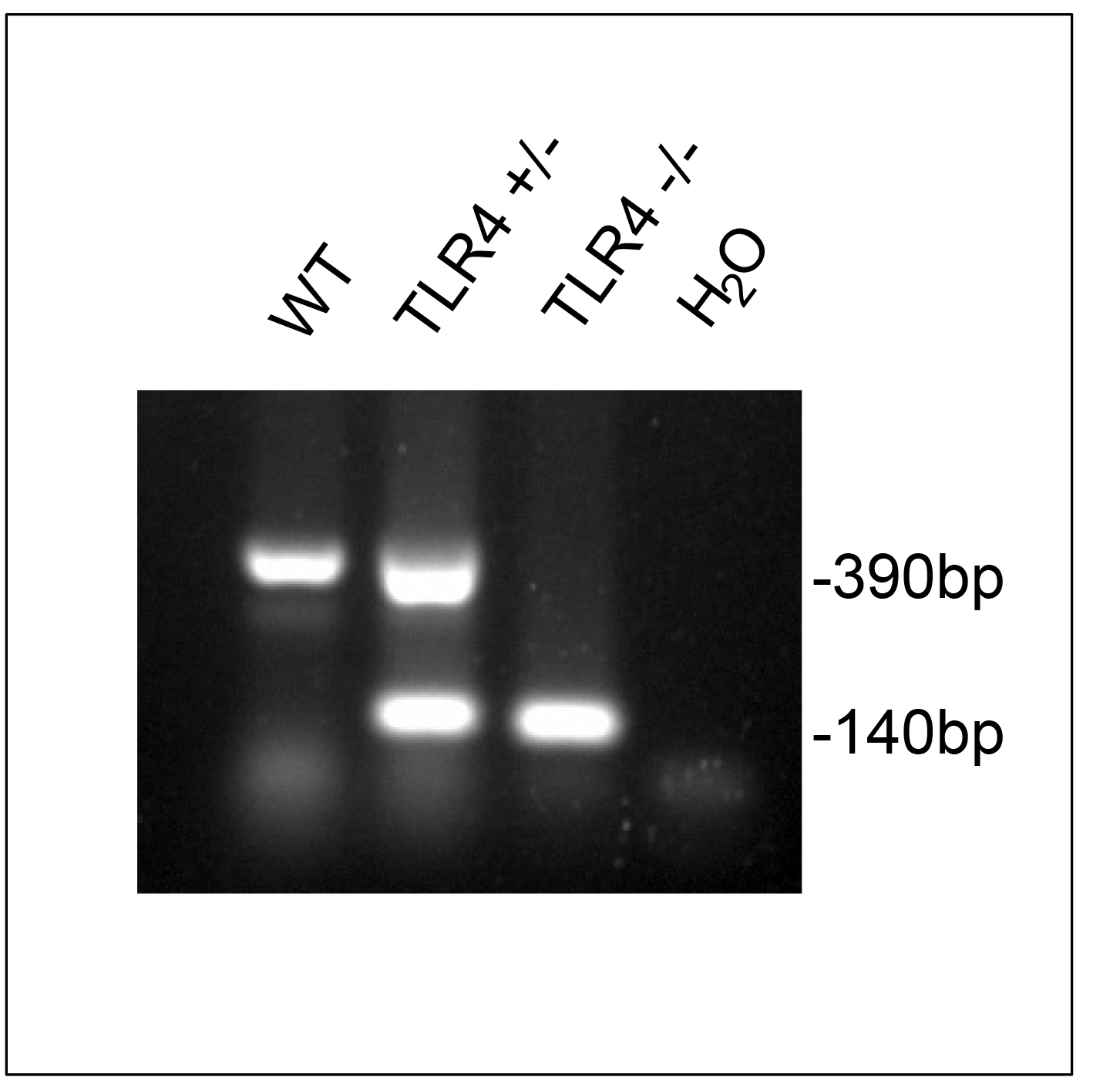

Supplement: Figure S1 — Genotyping of TLR4−∕− and WT mice. Offspring from the TLR4± intercross were genotyped. PCR amplification was carried out using tail genomic DNA from WT (lane 1), TLR4± (lane 2), and TLR4−∕− (lane 3) mice; H2O (lane 4) was used as a control. [file Image1.TIF]

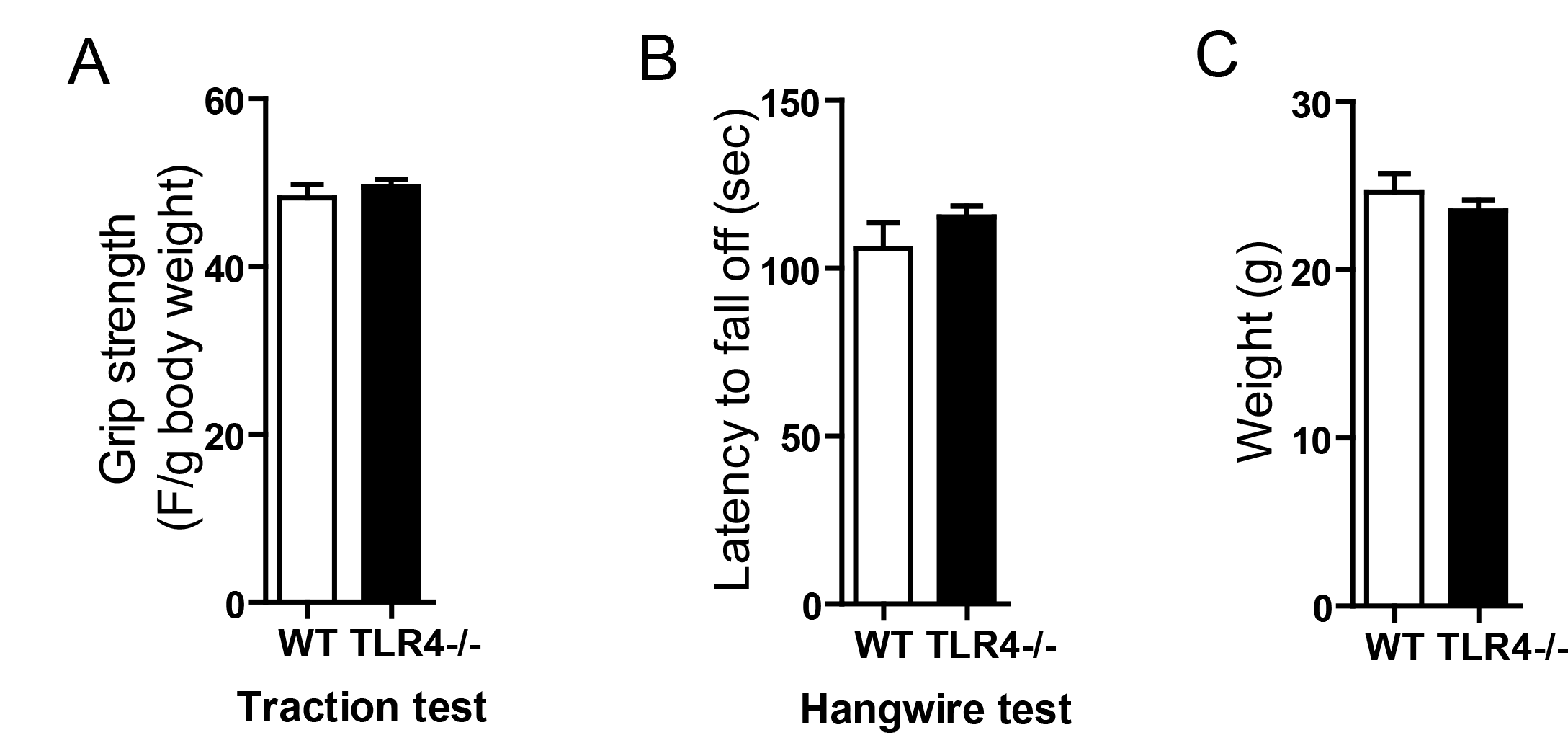

Supplement: Figure S2 — Loss of TLR4 has no effect on muscle strength. (A) TLR4−∕− and WT mice showed similar limb strength, as measured by the traction test. (B) Limb strength was evaluated with the hangwire test and was comparable between TLR4−∕− and WT mice. (C) TLR4 deficiency had no effect on body weight. Data are presented as mean ± SEM (n = 9–12 mice/group). [file Image2.TIF]

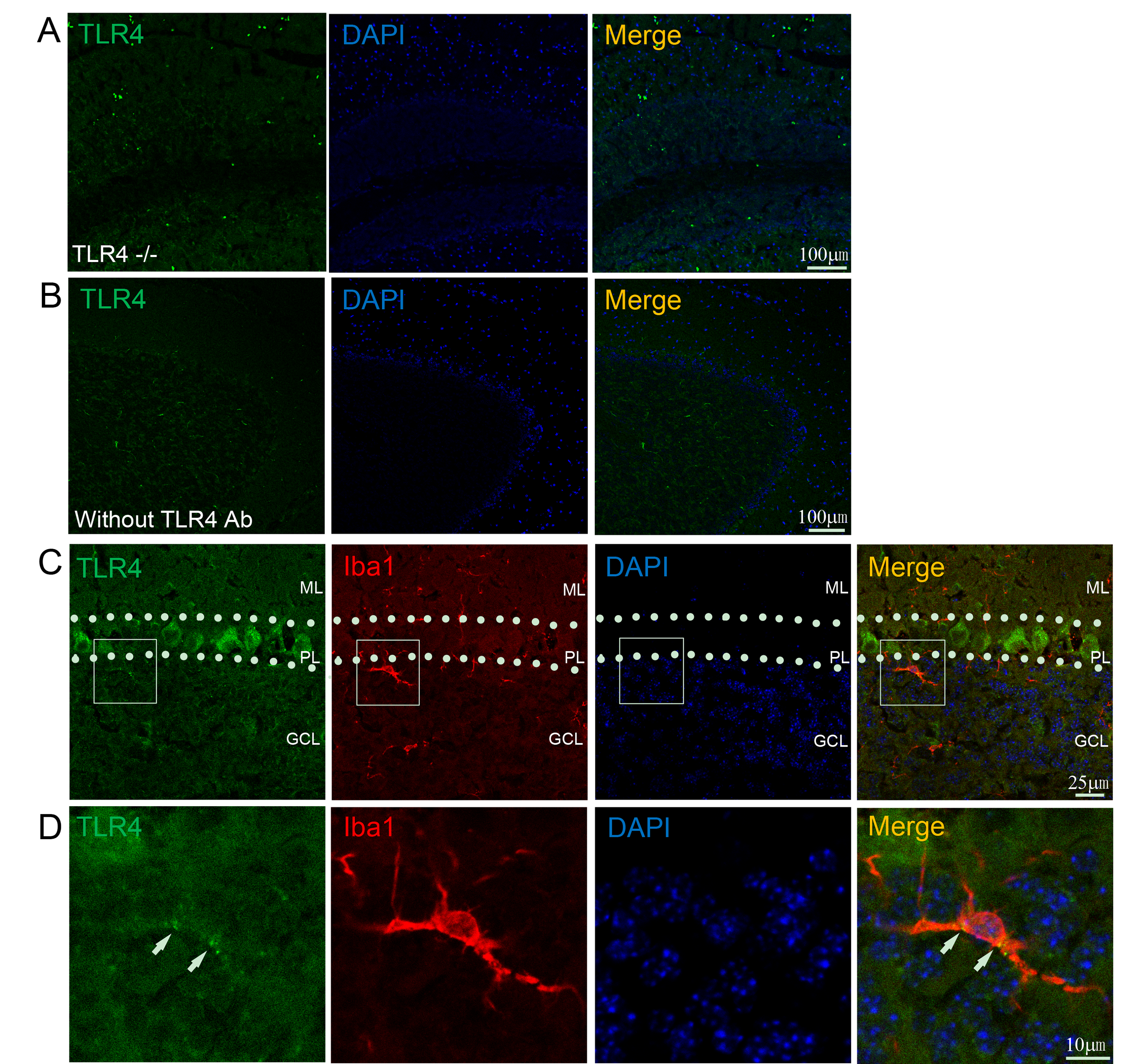

Supplement: Figure S3 — TLR4 is expressed in the PL of the cerebellum and in a small number of microglia. (A) Immunohistochemical analysis revealed no TLR4 expression in the PL of TLR4−∕− cerebella. (B) Weak fluorescence in other layers was attributed to background immunoreactivity. (C) TLR4 expression was also detected in Iba1-positive microglia, as shown by arrows in the high-magnification image (D). Scale bar = 100 μm in (A,B), 25 μm in (C), 10 μm in (D). TLR4 Ab, TLR4 antibody. [file Image3.TIF]

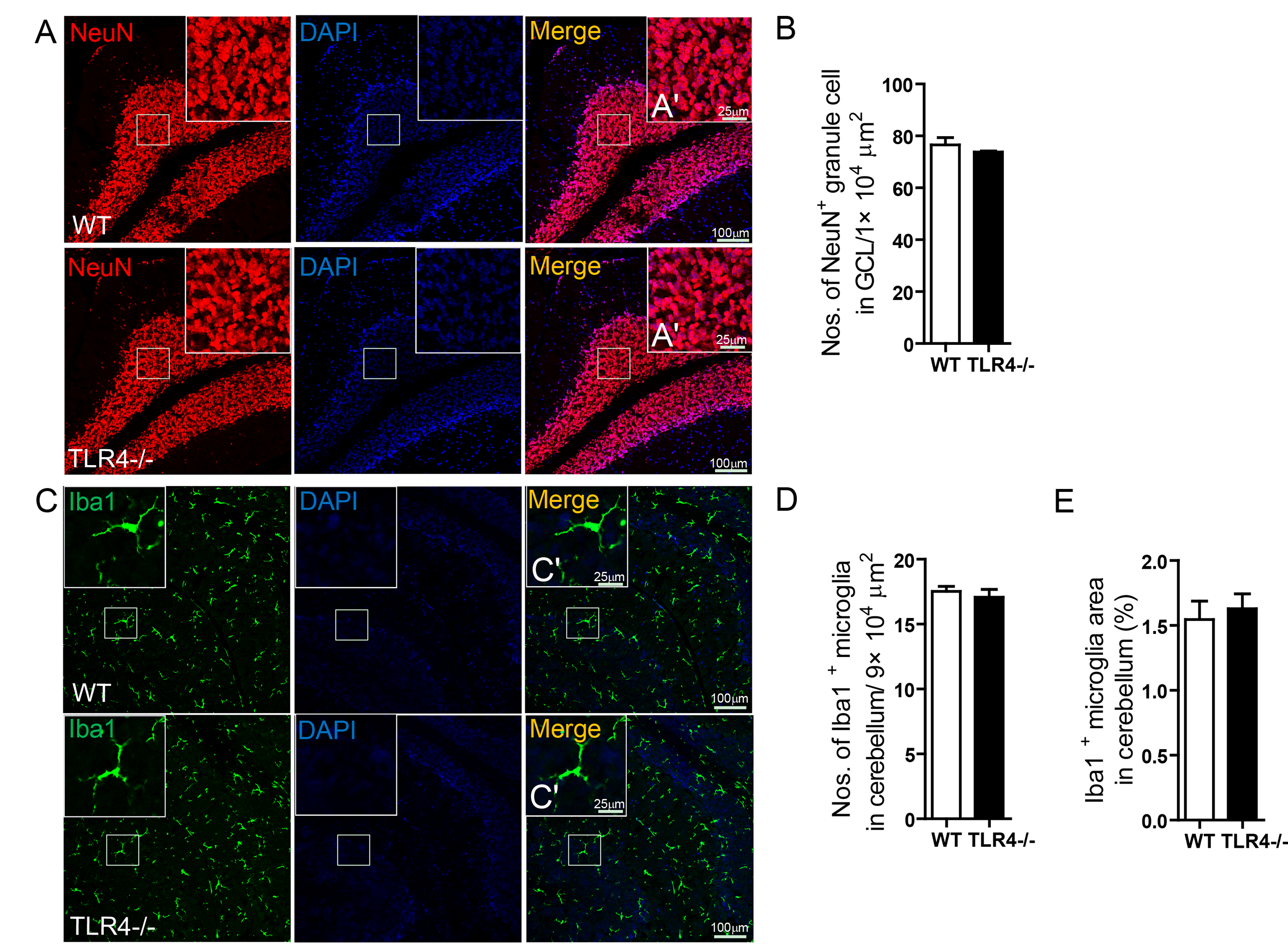

Supplement: Figure S4 — TLR4 deficiency has no effect on GC and microglia numbers. (A) NeuN-expressing GCs in the GCL, with a high-magnification image (A′). (B) Quantitative analysis of GC density revealed no difference between the two groups. (C) Iba1-positive microglia localized in the ML and GCL in both TLR4−∕− and WT mice. Microglia morphology was similar between the two groups, as shown in the high-magnification image (C′). (D,E) Quantitative analysis of cerebellar microglia showed no difference in microglia number (D) or percent area of microglia (E) between the two groups. Scale bar = 100 μm in (A,C), 25 μm in (A′,C′). Data are presented as mean ± SEM (n = 3 mice/group). [file Image4.TIF]
